# Neoadjuvant nivolumab with or without platinum-doublet chemotherapy based on PD-L1 expression in resectable NSCLC (CTONG1804): a multicenter open-label phase II study

**DOI:** 10.1038/s41392-023-01700-4

**Published:** 2023-12-06

**Authors:** Si-Yang Liu, Song Dong, Xue-Ning Yang, Ri-Qiang Liao, Ben-Yuan Jiang, Qun Wang, Xiao-Song Ben, Gui-Bin Qiao, Jun-Tao Lin, Hong-Hong Yan, Li-Xu Yan, Qiang Nie, Hai-Yan Tu, Bin-Chao Wang, Jin-Ji Yang, Qing Zhou, Hong-Rui Li, Ke Liu, Wendy Wu, Si-Yang Maggie Liu, Wen-Zhao Zhong, Yi-Long Wu

**Affiliations:** 1 Guangdong Lung Cancer Institute, Guangdong Provincial Key Laboratory of Translational Medicine in Lung Cancer, Guangdong Provincial People’s Hospital (Guangdong Academy of Medical Sciences), Southern Medical University, Guangzhou, China; 2 Department of Thoracic Surgery, Zhongshan Hospital, Fudan University, Shanghai, China; 3 Department of Thoracic Surgery, Guangdong Provincial People’s Hospital (Guangdong Academy of Medical Sciences), Southern Medical University, Guangzhou, China; 4 Department of Pathology, Guangdong Provincial People’s Hospital (Guangdong Academy of Medical Sciences), Southern Medical University, Guangzhou, China; 5 Fujian Key Laboratory of Advanced Technology for Cancer Screening and Early Diagnosis, Fuzhou, China; 6 Berry Oncology Corporation, Fuzhou, China; 7 Chinese Thoracic Oncology Group (CTONG), Guangzhou, China; 8 Department of Hematology, The First Affiliated Hospital, Jinan University, Guangzhou, China

**Keywords:** Lung cancer, Cancer therapy

## Abstract

This prospective multicenter phase II study evaluated the clinical efficacy of neoadjuvant nivolumab-exclusive (N) and nivolumab–chemotherapy (N/C) combinations based on PD-L1 expression. Eligible patients exhibited resectable clinical stage IIA–IIIB (AJCC 8th edition) NSCLC without *EGFR*/*ALK* alterations. Patients received either mono-nivolumab (N) or nivolumab + nab-paclitaxel+ carboplatin (N/C) for three cycles based on PD-L1 expression. The primary endpoint was the major pathological response (MPR). Key secondary endpoints included the pathologic complete response (pCR), objective response rate (ORR), and event-free survival (EFS). Baseline PD-L1 expression and perioperative circulating tumor DNA (ctDNA) status were correlated with pCR and EFS. Fifty-two patients were enrolled, with 46 undergoing surgeries. The MPR was 50.0% (26/52), with 25.0% (13/52) achieving pCR, and 16.7% and 66.7% for patients with PD-L1 ≥ 50% in N and N/C groups, respectively. Thirteen (25.0%) patients experienced grade 3 or higher immune-related adverse events during neoadjuvant treatment. Patients with post-neoadjuvant ctDNA negativity was more likely to have pCR (39.1%) compared with those remained positive (6.7%, odds ratio = 6.14, 95% CI 0.84-Inf, *p* = 0.077). With a median follow-up of 25.1 months, the 18-month EFS rate was 64.8% (95% CI 51.9–81.0%). For patients with ctDNA– vs. ctDNA + , the 18m-EFS rate was 93.8% vs 47.3% (HR, 0.15; 95% CI 0.04, 0.94; *p* = 0.005). Immunochemotherapy may serve as an optimal neoadjuvant treatment even for patients with PD-L1 expression ≥ 50%. ctDNA negativity following neoadjuvant treatment and surgery could help identify superior pathological and survival benefits, which requires further confirmation in a prospective clinical trial (NCT04015778).

## Introduction

Effective therapies are urgently required for early-stage non-small-cell lung cancer (NSCLC) patients. The cornerstone treatment for early-stage NSCLC is supposed to be surgery. However more than half of the patients experienced recurrence after the complete resection alone.^1^ As the standard treatment option for decades, postoperative platinum-based chemotherapy presents an absolute overall survival (OS) improvement of 5.4% at 5 years.^2–4^ Along with the limited survival benefits, such one-size-fits-all strategy may cause unnecessary adverse effects and psychological burdens. Precisely perioperative treatment with the accurate regimen and duration has become a vital challenge in this context.

The identification of oncogenic driver mutations and development of tyrosine kinase inhibitors (TKIs) have dramatically reinvented the management of advanced NSCLC. This innovative treatment concept as “individualized therapy” has continued to step into the perioperative setting. Matched targeted treatments have become optimal options in the perioperative setting for resectable NSCLC with oncogenic driver mutations.^5^ As for NSCLC without *EGFR*/*ALK* alternations, encouraged by the remarkable success of antibodies blocking the programmed death-1 (PD-1)/programmed death ligand-1 (PD-L1) immune inhibitory pathway in advanced stages, renewed interest has focused on revisiting neoadjuvant strategies.^6,7^ In the neoadjuvant context, immunotherapy can promote the early tumor-specific T cells expansion, induce significant adaptive anti-tumor responses, and eliminate micrometastases.^8–10^ Landmark studies have confirmed that neoadjuvant nivolumab alone can raise the pathologic complete response (pCR) up to 10% and the major pathological response (MPR) rates up to 22–45%.^11,12^ Chemotherapy is suggested to eliminate immuno-suppressive cells and induce tumor antigens exposure through tumor cell death induction in the tumor microenvironments; therefore, the role of neoadjuvant immunochemotherapy combinations has been further explored.^13^ The first phase III trial that compared nivolumab addition to neoadjuvant platinum-doublet chemotherapy, CheckMate-816, obtained a pCR of 24.3% and a statistically substantial event-free survival (EFS) rate improvement, with the risk of death or progression reduced by 37%.^14^ The subsequent single-arm phase II NADIM study further demonstrated the highly impressive pCR rate of 63.4% with neoadjuvant nivolumab plus chemotherapy followed by adjuvant 12-month nivolumab monotherapy especially in patients with resectable stage IIIA NSCLC.^15^ The pathological and survival benefits supplied by the perioperative immunochemotherapy design was effectively validated in the large randomized, double-blinded phase 3 CheckMate-77T study. With a minimum of 15.7-month follow-up, neoadjuvant immunochemotherapy reduced the risk of death or progression by 42% while pCR has been significantly improved to 25.3% compared with 4.7% by chemotherapy.^16^ Ever since then neoadjuvant nivolumab plus chemotherapy has become the most promising neoadjuvant treatment option for resectable NSCLC without *EGFR*/*ALK* alternations.

Although neoadjuvant nivolumab plus chemotherapy has already obtained the approval of the U.S. Food and Drug Administration for NSCLC treatment,^17^ head-to-head comparison of the efficacy of neoadjuvant immunotherapy-exclusive treatment and immunotherapy–chemotherapy combinations are currently lacking. Therefore, establishing predictive biomarkers is important for optimizing patient selection, guiding neoadjuvant monotherapy or combination regimens, and determining the optimal timing for surgery and adjuvant treatment escalation or de-escalation. In advanced-stage NSCLC, first-line anti-PD-1/PD-L1 monotherapy is favored for the patients with ≥ 50% pre-treatment PD-L1 expression.^18–21^ However studies on early neoadjuvant therapies have shown controversy on predictive values of PD-L1 expression. For instance, neoadjuvant atezolizumab alone achieved an MPR rate of 33% in patients with ≥ 50% PD-L1 expression, comparable with the 37% MPR rate achieved by immunochemotherapy.^14,22^ However, other studies of neoadjuvant mono-immunotherapy or immunochemotherapy have reported comparable responses in resectable NSCLC patients regardless of their PD-L1 expression.^12,14,23,24^ Therefore, the ability of PD-L1 expression in identifying patients that would potentially benefit most from immunochemotherapy or monotherapy remains unclear. Precise biomarkers to identify beneficial population are in urgent need. The success of precision medicine is dependent upon molecular profiling. Although the tumor tissue remains the “gold standard” for diagnosis and genomic sequencing, challenging biopsy locations, the limited availability of tissue for genotyping and spatial heterogeneity are significant innate limitations, especially in patients prior to the neoadjuvant treatment. The development of plasma circulating tumor DNA (ctDNA) detection offers a minimally invasive approach to capture the comprehensive genomic scope irrespective of tumor heterogeneity.^25^ Furthermore, ctDNA detection could signal the presence of minimal residual disease (MRD) status that may have the potential to serve as a real-time marker of postoperative recurrence and (neo)adjuvant therapy efficacy in NSCLC.^14,26^ Tracking serial ctDNA status could help identify patients who may have a higher risk of relapse ahead of imaging screening.^27^ Thus longitudinal ctDNA analysis during the whole treatment process might be able to locate patients who benefit from perioperative immunotherapy treatment in real-time.

In the current prospective, multicenter, phase II study, we examine the safety and efficacy of neoadjuvant nivolumab, with or without platinum-doublet chemotherapy, according to PD-L1 expression levels at the tumor baseline. We also profile the ctDNA status at post-surgery as well as pre-neoadjuvant and post-neoadjuvant treatment time points, then discuss the relationship between ctDNA dynamics and pathological responses and survival outcomes.

## Results

### Patients and treatment

Between 9 August 2019 and 5 August 2022, among the 54 patients evaluated for eligibility, 52 were enrolled in this study. Twelve (23.1%), 16 (30.8%), and 24 (46.2%) of the 52 patients were confirmed with < 1%, between 1% and 49%, and ≥ 50% PD-L1 expression, respectively. Twelve patients with ≥ 50% PD-L1 expression received neoadjuvant nivolumab-exclusive (N) and 40 patients received nivolumab–chemotherapy (N/C) combinations. All the patients underwent at least one-cycle neoadjuvant treatment. Among the prespecified arms, the disease characteristics and baseline demographics were generally well balanced (Table 1).

**Table 1:** Baseline characteristics

Parameter	A1 (%)	A2 (%)	B1 (%)	B2 (%)
Sex				
Male	8 (66.7)	10 (83.3)	14 (87.5)	10 (83.3)
Female	4 (33.3)	2 (16.6)	2 (12.5)	2 (16.7)
Age (median, range)	57 (29–71)	64 (43–78)	63 (22–73)	60.5 (48–72)
Performance status score				
0	2 (16.7)	0 (0)	1 (6.3)	2 (16.7)
1	10 (83.3)	12 (100)	15 (93.7)	10 (83.3)
Pathological type				
Adenocarcinoma	5 (41.7)	2 (16.6)	5 (31.3)	2 (16.7)
Squamous carcinoma	5 (41.7)	5 (41.7)	10 (62.5)	10 (83.3)
Adenosquamous	0 (0)	2 (16.6)	0 (0)	0 (0)
other	2 (16.6)	3 (25.0)	1 (6.2)	0 (0)
PD-L1 expression				
<1%	0 (0)	0 (0)	0 (0)	12 (100)
1–49%	0 (0)	0 (0)	16 (100)	0 (0)
≥ 50%	12 (100)	12 (100)	0 (0)	0 (0)
Clinical stage				
IIa	0 (0)	0 (0)	1 (6.2)	1 (8.3)
IIb	1 (8.3)	2 (16.6)	5 (31.3)	3 (25.0)
IIIa	7 (58.3)	9 (75.0)	8 (50.0)	6 (50.0)
IIIb	4 (33.3)	1 (8.3)	2 (12.5)	2 (16.7)
T stage				
1c	2 (16.6)	1 (8.3)	0 (0)	0 (0)
2a	1 (8.3)	2 (16.6)	4 (25.0)	2 (16.7)
2b	1 (8.3)	2 (16.6)	3 (18.8)	3 (25.0)
3	8 (66.7)	5 (41.7)	7 (43.8)	5 (41.7)
4	0 (0)	2 (16.6)	2 (12.5)	2 (16.7)
N stage				
0	0 (0)	0 (0)	3 (18.8)	2 (16.7)
1	3 (25.0)	8 (66.7)	8 (50.0)	6 (50.0)
2	9 (75.0)	4 (33.3)	5 (31.3)	4 (33.3)
M stage				
0	12 (100)	12 (100)	16 (100)	12 (100)

When the database was locked on August 5, 2022, 48 (92.3%) patients had fully completed the three-cycle neoadjuvant treatment, whereas 46 (88.5%) patients had undergone surgery and at least one-cycle adjuvant nivolumab treatment (Supplementary Fig. S1). Eighteen (39.1%) patients had completed the adjuvant treatment as per the protocol, 24 (52.2%) were still undergoing trial treatment, and four (8.7%) had discontinued the adjuvant nivolumab treatment. The median time length of the adjuvant nivolumab treatment was 50.6 weeks (range 2.9–52.1).

### Surgery summary

Forty-six (88.5%) of the 52 patients visited the operating room with surgery intentions. Among the six patients without surgery, one received nivolumab treatment (8.3%, 1/12) and five were treated in the nivolumab plus chemotherapy arm (12.5%; 5/40). The most common reasons for not undergoing surgery were toxicity (*n* = 3, 1.9%), disease progression (*n* = 2, 3.8%), and declined (*n* = 1, 1.9%). The percentages of patients with disease progression or adverse events were undifferentiated among the treatment groups (Table 2; Supplementary Fig. S1).

**Table 2:** Surgical outcomes

Postoperative evaluation	A1 (*n* = 12)	A2 (*n* = 12)	B1 (*n* = 16)	B2 (*n* = 12)
Patients with definitive surgery—no. (%)	11 (91.7)	10 (83.3)	14 (87.5)	11 (91.6)
Time from last neoadjuvant dose to definitive surgery—week, Median (IQR)	4 (4–6)	5 (4.8–5.3)	5 (4–5)	5 (4–5)
Patients with canceled definitive surgery—no. (%)	1 (8.3)	2 (16.7)	2 (12.5)	1 (8.3)
Disease progression	1 (100)	1 (50.0)	0 (0)	1 (100)
Adverse event	0 (0)	1 (50.0)	1 (50.0)	0 (0)
Other	0 (0)	0 (0)	1 (50.0)	0 (0)
Of patients with delayed surgery, proportion no. (%) with delay of ≤ 2 weeks	3 (27.2)	0 (0)	1 (7.1)	1 (9.1)
Surgical approach—no. (%)				
Thoracotomy	0 (0)	0 (0)	0 (0)	0 (0)
Minimally invasive	11 (100)	9 (90.0)	13 (92.9)	11 (100)
Minimally invasive to thoracotomy	0 (0)	1 (10.0)	1 (7.1)	0 (0)
Type of resection				
Lobectomy	10 (90.9)	6 (60.0)	12 (85.7)	11 (100)
Pneumonectomy	0 (0)	1 (10.0)	1 (7.1)	0 (0)
Other	1 (9.1)	3 (30.0)	1 (7.1)	0 (0)
Completeness of resection—no. (%)				
R0	10 (90.9)	10 (100)	14 (100)	11 (100)
R1	1 (9.1)	0 (0)	0 (0)	0 (0)
Median length of hospital stay—days (IQR)	9 (6–17)	10 (8–12)	8 (7–10)	8 (8–10)
Pathological lymph node evaluation				
N0	4 (36.4)	6 (60.0)	8 (57.1)	11 (100)
N1	1 (9.1)	3 (30.0)	5 (35.7)	0 (0)
N2	5 (45.4)	1 (10.0)	1 (7.1)	0 (0)
N3	1 (9.1)	0 (0)	0 (0)	0 (0)
Pathological lymph node downstage				
Yes	4 (36.4)	7 (70.0)	6 (54.5)	9 (100.0)
No	7 (63.6)	3 (30.0)	5 (45.5)	0 (0)

Of the 46 (100.0%) patients who were initiated using a minimally invasive (video-assisted thoracoscopic surgery) approach, only two (4.3%) were converted to thoracotomy. Forty-one (89.1%) patients underwent lobectomy, three (6.5%) underwent pneumonectomy, and one (2.2%) underwent sleeve lobectomy and wedge resection respectively. Forty-five (97.8%) patients underwent R0 surgical resection and 26 (56.5%) achieved pathological lymph node downstaging.

After the final dose of neoadjuvant treatment, the median interval to surgical resection was 32 days (range 26–56). Five surgeries (5/46, 10.9%) were delayed (Table 2). No surgical morbidity or mortality due to the neoadjuvant treatment was observed.

### Preliminary efficacy

**Fig. 1:**
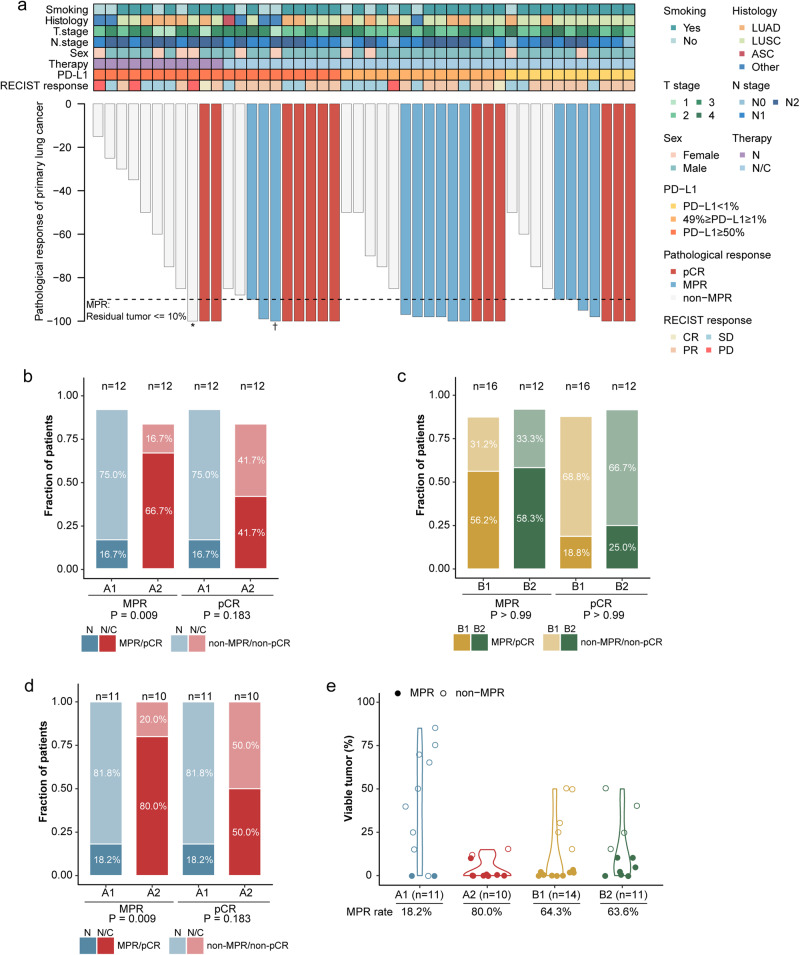
Pathological evaluation of responses to neoadjuvant nivolumab with or without chemotherapy on the basis of PD-L1 expression. **a** Pathological responses in resected primary lung tumors following neoadjuvant nivolumab treatment and nivolumab plus chemotherapy, based on the remaining viable tumor cells percentage, for each patient (*n* = 46) who received surgical resection. The black horizontal line represents the MPR threshold (90% regression). Clinical characteristics, pathological and preoperative radiologic responses are annotated for each patient. **b** Proportion of MPR/non-MPR and pCR/non-pCR in enrolled patients after neoadjuvant nivolumab and nivolumab plus chemotherapy (A1 & A2, PD-L1 ≥ 50%). **c** Proportion of MPR/non-MPR and pCR/non-pCR in enrolled patients after neoadjuvant nivolumab plus chemotherapy (B1, 1% ≤ PD-L1 ≤ 49%; B2, PD-L1 < 1%). **d** Proportion of MPR/non-MPR and pCR/non-pCR in resected patients following nivolumab plus chemotherapy and neoadjuvant nivolumab. **e** MPR/non-MPR in resected patients following nivolumab plus chemotherapy and neoadjuvant nivolumab based on PD-L1 expression. *p* values were calculated through Fisher’s exact tests. ASC adenosquamous carcinoma, CR complete response, LUAD adenocarcinoma, LUSC squamous-cell carcinoma, MPR major pathological response, N nivolumab monotherapy, N/C nivolumab plus chemotherapy, pCR pathologic complete response, PD progression disease, PD-L1 programmed death ligand-1, PR partial response, RECIST Response Evaluation Criteria in Solid Tumors Version 1.1, SD stable disease. * The preoperative PET-CT displayed an additional FDG uptake in the right supraclavicular lymph node then the patient underwent a wedge resection and the right supraclavicular lymph node sampling. No variable tumor in resected lung tissue but metastasis in the supraclavicular lymph node. † The patient received systematic mediastinal lymph node dissection and radical lobectomy. No variable tumor in primary tumor while metastasis in the hilar lymph node

For the intention-to-treat population, based on blinded independent pathological review (BIPR), the primary endpoint of MPR was 50.0% (26/52, 95% CI 35.8–64.2%), whereas the pCR rate was 25.0% (13/52, 95% CI 14.0–38.9%) (Fig. 1a). Among the patients with PD-L1 expression ≥ 50%, N/C induced a significantly higher MPR rate of 66.7% (A2, 8/12, 95% CI = 34.8–90.1%) than N (16.7%; A1, 2/12, 95% CI = 2.1–48.4%) (odds ratio = 14.85, 95% CI = 1.50–64.91, *p* = 0.009). Patients also more commonly obtained pCR after N/C (41.7%; 5/12, 95% CI 15.2–72.3) than after N (16.7%; 2/12, 95% CI 2.1–48.4%) (Fig. 1b). For PD-L1 expressions of 1–49% (B1) and < 1% (B2), the MPR rate after neoadjuvant N/C was 56.3% (B1, 9/16, 95% CI = 29.9–80.2%) and 58.3% (B2, 7/12, 95% CI = 27.7–84.8%), and the pCR rate was 18.8% (B1, 3/16, 95% CI = 4.0–45.6%) and 25.0% (B2, 3/12, 95% CI = 5.5–57.2%), respectively (Fig. 1c).

As resected specimens were not available for six patients who received treatment without undergoing surgery, a sensitivity analysis was carried out to examine the MPR and pCR rates in those who underwent surgical resection. Among the 46 patients resected in the trial, a significantly higher incidence of MPR in the N/C group was observed than that in the N group among the patients with ≥ 50% PD-L1 expression (80.0% vs.18.2%, odds ratio = 14.85, 95% CI 1.50–64.91, *p* = 0.009). pCR was also more common in the N/C group (50.0% vs.18.2%, odds ratio = 4.17, 95% CI 0.46–59.60, *p* = 0.183) (Fig. 1d). In the post-hoc analysis, no significant correlations between the pathological responses and the baseline PD-L1 expression were identified in the patients who received neoadjuvant immunochemotherapy (MPR, A2 vs. B1 vs. B2, 80.0% vs. 64.3% vs. 63.6%, *p* = 0.428) (Fig. 1e).

In all treated populations, the overall response rate (ORR) was 55.8% (29/52, 95% CI 41.3–69.5%), whereas in the N group and N/C group, the ORRs were 41.7% (5/12, 95%CI 15.2–72.3%) and 60% (24/40, 43.3–75.1%), respectively (Supplementary Table S1 and Table S2). Additionally, two patients that achieved stable disease radiographically were assessed as pCR after surgery.

**Fig. 2:**
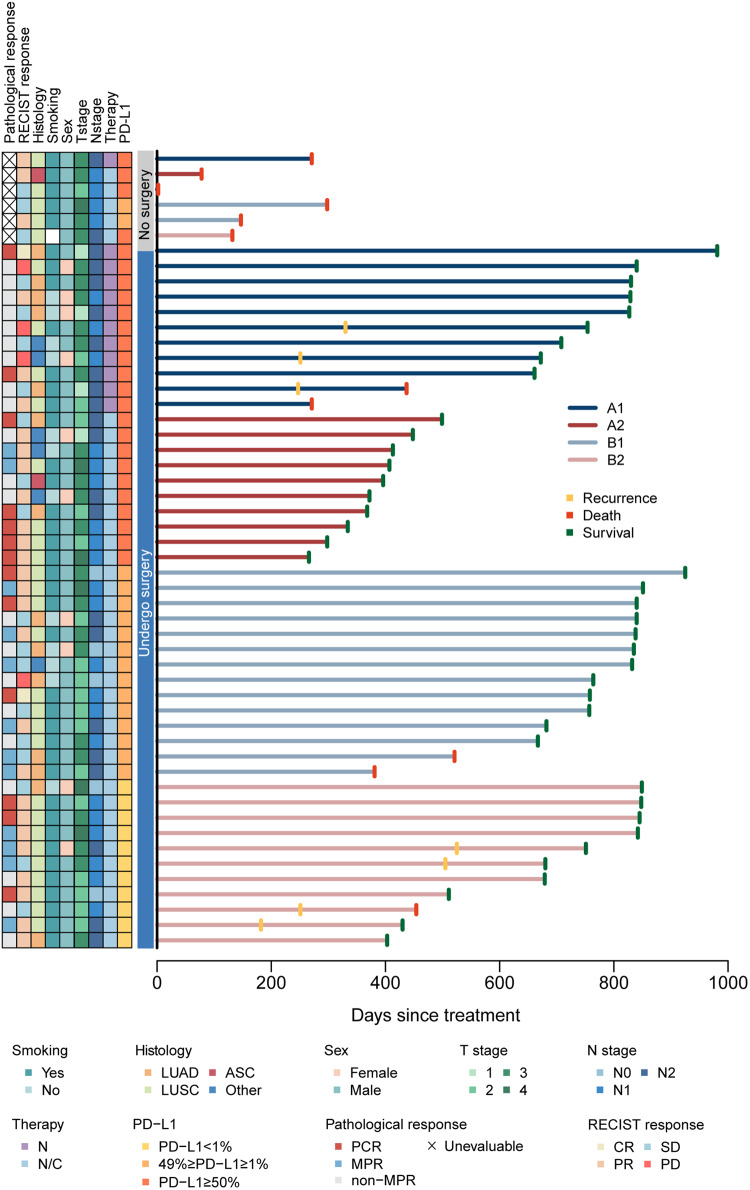
Swimmer plot depicting events in all enrolled population (*n* = 52). The left column displays pathological responses and clinical characteristics of the patients. Each bar represents one patient. Seven patients (13.5%) who received surgery had experienced disease progression, and two (3.8%) of them had died. ASC adenosquamous carcinoma, LUAD adenocarcinoma, LUSC squamous carcinoma, N nivolumab monotherapy, N/C nivolumab plus chemotherapy, RECIST Response Evaluation Criteria in Solid Tumors Version 1.1

With a median follow-up of 25.1 months for the entire enrolled population (95% CI 22.0–27.7 months, range 0.07–32.7 months), neither the median EFS nor the median OS was reached. Nine (17.3%) of the 52 patients experienced disease progression or died, two of whom (3.8%) did not undergo surgery (Fig. 2). Thirty-six (78.6%) of the 46 patients with tumor resection were recurrence-free and alive, with an 18-month EFS rate of 64.8% (95% CI 51.9–81.0%). As the median 12.4-month follow-up in A2 was relatively shorter than the other three arms which was 27.6, 27.6, and 25.1 months in A1, B1, and B2, respectively, survival outcomes might need to be interpreted cautiously.

### Safety

Overall, adverse events were manageable, without new concerns of safety in comparison to the known safety profiles for neoadjuvant nivolumab monotherapy and nivolumab plus chemotherapy. Of the 52 patients in the safety-evaluable population, 36 (69.2%) experienced at least one immune-related adverse event during neoadjuvant treatment, and 13 (25.0%) experienced grade ≥ 3 adverse events. Exhaustion (40.3%), appetite loss (32.7%), and anemia (28.8%) were among the most common grade 1/grade 2 immune-related adverse events. White blood cell count reduction (16.7%, *n* = 2) and neutrophil count reduction (10.0%, *n* = 4) were the most frequent grade ≥ 3 immune-related adverse events among the 12 patients receiving N and the 40 patients receiving N/C, respectively. Only one patient death among the three (5.8%) who died in the neoadjuvant phase was related with immune treatment (pneumonitis) (Supplementary Table S3).

### Analysis of ctDNA

**Fig. 3:**
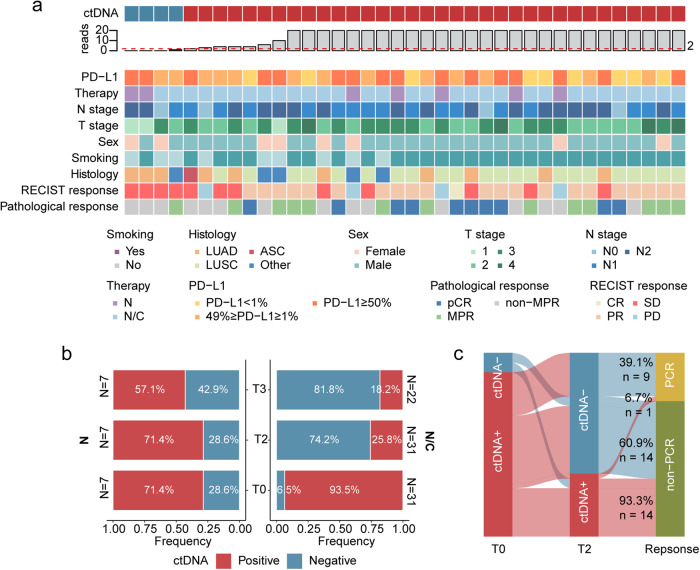
Associations between circulating tumor DNA (ctDNA) and responses to neoadjuvant treatment. **a** Patients and tumor characteristics based on the baseline ctDNA status (T0). **b** ctDNA dynamics during neoadjuvant nivolumab monotherapy and nivolumab plus chemotherapy. Proportion of patients based on ctDNA positivity according to available numbers of samples per time point. **c** Sankey plot displaying ctDNA dynamics (clearance/non-clearance) during neoadjuvant treatment versus response (pCR/non-pCR). Analyses were carried out for the patients with positive ctDNA at T0 (baseline) and corresponding ctDNA test results at T2 (following completing neoadjuvant therapy). N nivolumab monotherapy, N/C nivolumab plus chemotherapy, pCR pathologic complete response

The collected plasma samples at the pre-treatment period were analyzed in 38 patients. ctDNA was detected in 89.5% (34/38) of pre-treatment samples overall, including 21.1% (8/38) with stage II disease and 68.4% (26/38) with stage III disease (Fig. 3a, Supplementary Table S4).

The ctDNA detection rate decreased during neoadjuvant treatment, from 89.5% before treatment (T0) to 34.2% after neoadjuvant treatment (T2), then continued to decrease to 27.6% after surgery (T3). Specifically, neoadjuvant N and N/C reduced ctDNA positivity by 42.9% (T3) and 18.2% (T3), respectively (Fig. 3b and Supplementary Table S5).

**Fig. 4:**
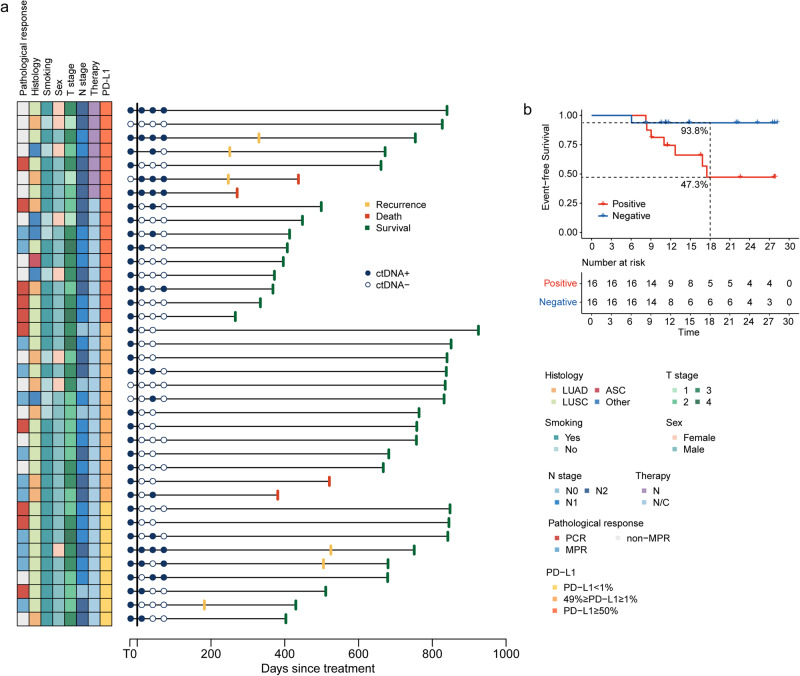
Circulating tumor DNA (ctDNA) and survival outcomes. **a** Swimmer plot depicted events in patients with baseline ctDNA testing (*n* = 38). The left column displays pathologcial responses and clinical characteristics. Each bar represents one patient. **b** Patient survival according to ctDNA/MRD status at T2 (after the completion of neoadjuvant treatment) and T3 (1 month after surgery, prior to adjuvant treatment). Negative, ctDNA/MRD, assessed as negativity at both T2 and T3; Positive, ctDNA/MRD+, assessed as positivity at either T2 or T3. ASC adenosquamous carcinoma, LUAD lung adenocarcinoma, LUSC lung squamous carcinoma, MPR major pathological response, N nivolumab monotherapy, N/C nivolumab plus chemotherapy, pCR pathologic complete response

We next evaluated whether ctDNA clearance was associated with pCR rates. Among the patients with detectable ctDNA at T0 and underwent the corresponding T2 plasma measurements, 23 were ctDNA-negative at T2, with a ctDNA clearance rate of 67.6% (23/34) in the whole cohort. The percentage of patients with ctDNA clearance who received neoadjuvant N/C was significantly higher than those who received N (75.8% vs. 20%; odds ratio = 11.49, 95%CI 0.94–64.36, *p* = 0.029). As much as 39.1% of patients with undetectable ctDNA at T2 were without residual disease at surgery (pCR 9/23), in comparison to 6.7% of patients with detectable ctDNA at T2 (pCR 1/15) (Fig. 3c), showing a substantial association (odds ratio = 6.14, 95%CI = 0.84-Inf, *p* = 0.077). With a median 22.7-month follow-up (95%CI = 22.0–27.7 months, range 8.9–30.8 months) for all 38 patients, four experienced local recurrence and two experienced distant metastases, one of whom died (Fig. 4a). Notably, detectable ctDNA was observed at a minimum of one point in all patients with local and distant recurrences. Interestingly, the 18-month EFS rate for patients with ctDNA/MRD– (both T2 and T3) vs. those with ctDNA/MRD+ (either T2 or T3) was 93.8% vs. 47.3% (HR, 0.15; 95% CI: 0.04, 0.94; *p* = 0.005) (Fig. 4b).

## Discussion

Our study is the first prospective investigator-initiated study examining the roles of PD-L1 expression in neoadjuvant N and N/C efficacy. Both the prespecified subgroups, with an overall 50% MPR rate, met the primary endpoint. Our results further demonstrated that, even in resectable NSCLC with PD-L1 expression ≥ 50%, neoadjuvant N resulted in a significantly lower MPR rate than N/C (16.7% vs. 66.7%). Other key outcomes, including radiographic objective responses and pathological complete responses, also favored N/C, regardless of PD-L1 expression at the tumor baseline. In further exploratory analysis, ctDNA clearance prior to surgery was also more common among the patients receiving N/C and was significantly associated with pCR. Moreover, the continued reduction in postoperative ctDNA detection indicated the necessity of surgery as well.

As the goal of early-stage NSCLC treatments is curing the disease, one of the main objectives of neoadjuvant treatments is to increase resectability. Therefore, radiographic responses are crucial for evaluating efficacy owing to their critical correlation with surgical feasibility. The other main goal should be to elevate MPR and/or pCR, which has been considered a promising predictor of survival benefits.^28^ Herein, the greater ORR induced by neoadjuvant immunochemotherapy, combined with superior MPR and pCR in our study, indicated that nivolumab plus chemotherapy is an effective neoadjuvant regimen.

Surgical outcomes were also acceptable with immunochemotherapy, with greater use of minimally invasive surgery, fewer surgery cancellation (including disease progression), and less pneumonectomy cases, which is a type of surgery typically characterized by trauma and a poorer prognosis. In the CheckMate-816 trial, only 30% of enrolled patients underwent minimally invasive surgery, whereas 95.7% underwent this procedure in this study. This is similar to our previous neoadjuvant immunotherapy trial, which showed that minimally invasive surgery is feasible after neoadjuvant immunochemotherapy.^29,30^

The safety profile was in accordance with that of previous reports. The onset of only one case of grade 5 immune-mediated pneumonitis occurred one cycle after neoadjuvant immunochemotherapy, despite optimal medical management. Moreover, nivolumab did not hinder the surgery feasibility or increase surgery-related adverse events when added to neoadjuvant chemotherapy. Overall, considering its safety, surgical feasibility, and efficacy, nivolumab combined with chemotherapy is a rational choice for the neoadjuvant setting.

However, no associations between the pathologic response and the baseline PD-L1 expression in patients receiving immunochemotherapy were observed, being consistent with those found in the NADIM study.^15^ Thus, real-time predictive biomarkers are required to identify beneficial populations, maximize therapeutic benefit, and minimize the risk of toxicities from neoadjuvant immunotherapy.

Perioperative ctDNA detection is minimally invasive to detect MRD and relapse risk stratification of early-stage NSCLC.^31–35^ Longitudinal undetectable MRD represents a possibly cured population after curative surgery and indicate the non-essentiality of adjuvant therapy.^36,37^ Hence ctDNA-MRD monitoring may benefit NSCLC patient management in adjuvant de-escalation treatment. ctDNA studies have been conducted in the neoadjuvant setting,^14,26^ with ctDNA clearance associated with higher pCR rates and longer EFS in the CheckMate-816 trial. Furthermore, low pre-treatment levels of ctDNA in the NADIM trial acted as a prognostic biomarker for improved PFS and OS. However, neither of the above studies explored the MRD status after surgery.

In this study, post-treatment ctDNA status was significantly correlated with pathological responses. ctDNA clearance prior to surgery was also more common among the patients receiving immunochemotherapy, and was significantly associated with pCR. As pCR is a promising early indicator of survival efficacy in the patients with resectable NSCLC, ctDNA clearance during neoadjuvant therapy might serve as an early candidate predictor for favorable outcomes. Additionally, patients with ctDNA negativity at both post-neoadjuvant and post-surgery time points achieved a significantly higher 18-month EFS rate than patients who remained ctDNA-positive at either time point. Thus, the combined detection of MRD after the completion of neoadjuvant treatment and surgical practices might assist in identifying the patients who potentially have already been cured and do not require a 12-month-course adjuvant therapy.

We acknowledge that the current study is associated with limitations including its relatively short follow-up duration and relatively small sample size. Regarding neither the median EFS nor OS was reached, the interpretation of the survival outcomes might be limited. Additionally, a longer follow-up period is essential for capturing the clinical benefits of post-neoadjuvant ctDNA clearance and post-surgery MRD status in patients with a more favorable prognosis. We have since expanded this study to include perioperative monitoring of ctDNA focusing on prediction of recurrences and detection of residual diseases. Longitudinal plasma samples were also prospectively collected to assess post-surgical MRD status. Data on longitudinal MRD detection was exploratory and will be presented elsewhere. It should be noted that, owing to the limited MPR rate of 18.2% induced by neoadjuvant nivolumab alone, we merely recommend neoadjuvant nivolumab combined with chemotherapy in the expanded cohort (NCT04015778). If validated, dynamic evaluations of ctDNA by liquid biopsy might facilitate individualize perioperative treatment to maximize the curing possibility and avoid the risk of overtreatment.

In conclusion, neoadjuvant immunochemotherapy is a standard therapeutic approach for patients with resectable NSCLC. Moreover, ctDNA analysis at both post-surgery and post-neoadjuvant treatment time points could help identify superior pathological responses and survival benefits, which requires a prospective clinical trial (NCT04015778) for further confirmation.

## Patients and methods

### Study design

As an open-label, multicenter, phase II clinical trial, this study evaluated the clinical activity and safety of neoadjuvant nivolumab-exclusive and nivolumab–chemotherapy combinations for resectable NSCLC based on the tumor expression of PD-L1 at the baseline (NCT04015778). The study was conducted in accordance with the Declaration of Helsinki and the Good Clinical Practice, and approved by the clinical research ethics committee of each participating center. All patients provided written informed consents prior to enrollment. The full study protocol is provided as a supplementary material.

### Patients’ eligibility

Eligible adults had cytologically or histologically confirmed stage IIA–IIIB NSCLC (according to 8th edition American Joint Committee on Cancer criteria), with a 0 or 1 score of Eastern Cooperative Oncology Group performance status, previously had not underwent any systemic therapy, and were suitable for definitive resection. Patients were required to have measurable disease based on the Response Evaluation Criteria in Solid Tumors (RECIST) version 1.1, as well as pre-treatment tumor tissues for assessing PD-L1 expression. Positron emission tomography/computed tomography (PET/CT) scan was mandatory for each patient inclusion. Pathological evaluations of mediastinal lymph nodes at levels 4 (bilaterally) and 7 by thoracotomy, mediastinoscopy, or endobronchial ultrasound was required for clinical staging of patients with mediastinal adenopathy on PET/CT. Patients with sensitizing *EGFR* or *ALK* alteration evaluated at any study site were excluded.

### Treatment

Patients with ≥ 50% PD-L1 expression were assigned for neoadjuvant nivolumab monotherapy (360 mg, on day 1) intravenously (N) for three cycles (each 21-day cycle) (arm A1) at first. After prospectively completing arm A1 enrollment, the following patients with ≥ 50% PD-L1 expression were enrolled in arm A2 with nivolumab (360 mg, on day 1) plus nab-paclitaxel (135 mg/m^2^, on days 1 and 8) plus carboplatin (area under the curve five, on day 1) (N/C) for three cycles (each 21-day cycle). For PD-L1 expression of 1–49% (arm B1) and < 1% (arm B2), patients were assigned for immunochemotherapy, following the same regimen as mentioned above.

Surgery was planned within 42 days following completing the neoadjuvant treatment, after which the patients received 12-month adjuvant intravenous nivolumab monotherapy (360 mg once every 3 weeks) (Supplementary Figure S1). The primary tumor and lymph nodes were resected following standard institutional procedure.

### Endpoints and assessments

The primary endpoint was the MPR (i.e., ≤ 10% viable tumor cells in the primary tumor at resection based on a BIPR). The MPR was evaluated locally according to standard operating procedure and study-specific pathology training.

Secondary endpoints included the MPR according to PD-L1 expression levels, investigator-assessed ORR based on RECIST, pCR (i.e., no residual viable tumor cells in the primary tumor and sampled lymph nodes based on BIPR), pathological lymph node downgrade rate, EFS, OS, and treatment toxicity and surgery feasibility measured by the incidence of immune-related adverse event. Indicators for surgery feasibility included the completeness of resection, operative approach and type, mortality, morbidity, and complications within the first 90 days following the surgery. Treatment toxicity was evaluated for 100 days following the last dose of adjuvant nivolumab or neoadjuvant, based on NCI-CTCAE (version 4.0) guidelines.

### Correlative analysis

Correlative analysis to explore potential biomarkers, included PD-L1 expression and paired-exome sequencing of blood and tumor ctDNA.

The PD-L1 status was immunohistochemically examined by the DAKO PD-L1 (28-8 pharmDx) assay. ctDNA was analyzed using a tumor-informed ctDNA panel for targeted gene sequencing. Briefly, patients with tumor tissues available were sequenced at first for specific tumor variants identification. Two distinct methods were employed to call plasma ctDNA variants depending on if they were identified in matched tumor tissues. The detailed description of plasma ctDNA analysis is available in the Supplementary Methods.

Plasma samples collection was carried out at pre-treatment (T0), prior to the third cycle of neoadjuvant treatment (T1), after neoadjuvant treatment prior to surgery (T2), and within one month after surgery (T3, prior to adjuvant treatment). ctDNA clearance was defined as a pre-surgery alteration from detectable ctDNA at T0 to undetectable ctDNA at T2.

### Statistical analysis

Based on the study design, the patients were separated into arms A and B according to PD-L1 expression. Assuming an MPR rate of 35%, based on the literature and a 5% dropout rate, a sample size of 24 patients at least in each arm was employed to obtain 80% power with a type I error of 0.025.^4,38–40^ To further provide insights into PD-L1 guidance on distinct neoadjuvant regimens, arm A was divided into subgroups A1 (*n* = 12) and A2 (*n* = 12), which received neoadjuvant nivolumab-exclusive (N) treatment or nivolumab–chemotherapy (N/C), respectively, whereas arm B was divided into subgroups B1 (*n* = 16, planned to enroll 12 cases while actually enrolled 16 cases) and B2 (*n* = 12) according to 1–49% or < 1% PD-L1 expression, respectively.^41,42^

The efficacy and safety analyses set were evaluated in all enrolled patients, and the 95% confidence interval (CI) for response rates were calculated utilizing the exact binomial distribution. Qualitative variables are presented as the absolute and relative frequency, and quantitative variables are presented as the median (IQR) or mean (SD).

Associations between qualitative variables were tested through the Cochran–Armitage and Fisher’s exact tests. Survival curves were generated through Kaplan–Meier analysis and compared through the log-rank tests. Hazard ratios (HR) and the associated 95% CIs were calculated based on the Cox proportional hazard models. All statistical analyses were carried out utilizing R statistical software (version 3.6.2).

### Supplementary information

Supplementary Information

## Data Availability

All relevant data that support the findings of this study are available upon reasonable request from the corresponding author (Y.-L.W., syylwu@live.cn).
