# Supplementary material for: Neoadjuvant nivolumab with or without platinum-doublet chemotherapy based on PD-L1 expression in resectable NSCLC (CTONG1804): a multicenter open-label phase II study
Source: Signal Transduct Target Ther. 2023 Dec 6;8:442. doi: 10.1038/s41392-023-01700-4 (PMC10700550; doi:10.1038/s41392-023-01700-4)

**Supplementary Materials for**

**Neoadjuvantnivolumab with or without platinum-doublet chemotherapy based on**

**PD-L1 expression in resectable NSCLC (CTONG1804):**

**A multicenter open-label phase II study**

Si-Yang Liu#1, Song Dong#1, Xue-Ning Yang#1, Ri-Qiang Liao1, Ben-Yuan Jiang1, Qun Wang2, Xiao-Song Ben3, Gui-Bin Qiao3, Jun-Tao Lin1, Hong-Hong Yan1, Li-Xu Yan4, Qiang Nie1, Hai-Yan Tu1, Bin-Chao Wang1, Jin-Ji Yang1, Qing Zhou1, Hong-Rui Li5,6, Ke Liu5,6, Wendy Wu5,6, Si-Yang Maggie Liu7, 8, Wen-

Zhao Zhong*1, Yi-Long Wu*1

Correspondence to: Prof. Wen-Zhao Zhong at syzhongwenzhao@scut.edu.cn & Professor Yi-Long Wu at syylwu@live.cn; Affiliation: Guangdong Lung Cancer Institute, Guangdong Provincial People’s Hospital (Guangdong Academy of Medical Sciences), Southern Medical University, Address: 106 Zhongshan 2nd Rd, Guangzhou, Guangdong, China, 510080. Tel: +8620 83877855

**This PDF file includes:**

Methods

Tables S1 to S6

Figures. S1 to S2

**TABLE OF CONTENTS**

**1. Supplementary Methods** [1](#bookmark1)

**2. Supplementary Table** [3](#bookmark2)

Table S1 The overall response rate in the intention to treat population (n, %) .......3

Table S2 The overall response rate in theresected population (n, %) [4](#bookmark3)

Table S3. Incidence of Immune-mediated Adverse Events associated with

neoadjuvant therapy (Safety Population) † [5](#bookmark4)

Table S4 Characteristics of patients underwent ctDNA analysis [7](#bookmark5)

Table S5 ctDNA/MRD status at each timepoint [9](#bookmark6)

**3. Supplementary Figures** [10](#bookmark7)

Figure S1 Patient Disposition [10](#bookmark8)

Figure S2 Trial schema [11](#bookmark9)

**1. Supplementary Methods**

*Tissue genomic DNA extraction and plasm DNA isolation*

Tumor tissues were fixed with formalin, subsequently embedded in paraffin (FFPE). Genomic DNA (gDNA) was extracted from each FFPE sample using the QIAamp DNA FFPE Tissue Kit (Qiagen, Hilden, Germany) according to the manufacturer’s protocol, as well as, from the white blood cell samples using MagPure Tissue & Blood DNA LQ Kit (Magen, Guangzhou, China) according to the manufacturer’s instructions,

respectively.

The plasma cell free DNA (cfDNA) was isolated using QIAamp Circulating Nucleic Acid Kit (Qiagen, Hilden, Germany) according to the manufacturer's protocol. The quality of purified DNA was assayed by gel electrophoresis and quantified by Qubit RNA HS Assay

Kit (Invitrogen, Carlsbad, USA).

*gDNA Library constructions and bioinformatics analysis*

gDNA library was constructed using CS2.0 Tissue DNA Library Prep Kit (Berry Oncology, Fujian, China) according to manufacturer’s instructions. To construct the pre-library of gDNA, genomic DNA was digested into ~200 bp fragments by enzymatic method, then subjected to end repairing, A-tailing, adapter ligation and universal amplification as previously reported 1. Purified pre-library was hybridized with a customized biotin probe pool (the 654 genes panel, Berry Oncology, Fujian, China) to capture target fragments. Captured fragments were amplified with universal primers and purified to acquire the final library. The library of pairended multiplex samples were sequenced with the NovaSeq 6000 System. Sequencing depth was _2,000 x per sample. The generated sequences were trimmed, low-quality-filtered, and subjected for variant calling. Variants was filtered for nonsynonymous SNPs, indels and spliced variants. Somatic variations were identified with variant allele frequency (cutoff _ 3%) and cancer hotspots were screened with variant allele frequency (cutoff _ 1%) and at least

20 high-quality reads.

*cfDNA library constructions and bioinformatics analysis*

For targeted sequencing of cfDNA, the pre-libraries were prepared according to the

method previously described 2. cfDNA library was constructed using CS2.0 cfDNA Library Prep Kit (Berry Oncology, Fujian, China) according to manufacturer’s instructions. Amplified libraries were enriched for genes regions of interest by liquid hybridization using CS2.0 DNA Hybridization and Wash Kit (Berry Oncology, Fujian, China). An in-house designed panel was used to capture cfDNA fragments to generate sequencing library. The sequencing libraries were applied on NovaSeq 6000 platform

with 150PE mode for the average depth of plasma samples > 15000X.

The generated sequences were trimmed, low-quality-filtered, and subjected for variant calling. Based on whether the plasma variants were identified in matched tumor tissue, two different methods were used to define plasmactDNA variants. For tissue-derived variants in plasma, the variants with ≥ 3 high-quality support reads were considered as positive. For cfDNA variants not occurring in matched tumor tissue, if the following stringent conditions were met, they were considered to be positive: (i) for hotspot mutations, ≥ 0.1% mutation frequency and ≥ 3 high-quality support reads; and (ii) for non-hotspot mutations, ≥ 0.5% mutation frequency and ≥ 5 high-quality support reads. Clonal hematopoiesis were filtered through deep sequencing of paired

white blood.

**References**

1 Cheng, Y. *et al.* The Comprehensive Analyses of Genomic Variations and

Assessment of TMB and PD-L1 Expression in Chinese Lung Adenosquamous Carcinoma. *Frontiers in genetics* **11**, 609405 (2020).

2 Lv, W. *et al.* Noninvasive prenatal testing for Wilson disease by use of circulating single-molecule amplification andresequencing technology (cSMART). *Clinical chemistry* **61**, 172-181 (2015).

**2. Supplementary Table**

**Table S1 The overall response rate in the intention to treat population (n, %)**

|  | A1 (12) | A2 (12) | B1 (16) | B2 (12) |
| --- | --- | --- | --- | --- |
| CR | 1 (8.3) | 0 (0) | 1 (6.3) | 0 (0) |
| PR | 3 (25.0) | 7 (58.3) | 7 (43.8) | 8 (66.7) |
| SD | 4 (33.3) | 3 (25.0) | 6 (37.5) | 3 (25.0) |
| PD | 4 (33.3) | 0 (0) | 1 (6.3) | 1 (8.3) |
| NA | 0 (0) | 2 (16.7) | 1 (6.3) | 0 (0) |
| ORR | 4 (33.3) | 7 (58.3) | 8 (50.0) | 8 (66.7) |

CR, complete response; PR, partial response; SD, stable disease; PD, progressive disease; NA, notevaluable; ORR,a complete or partial response from baseline to the

presurgery scan per Response Evaluation Criteria in Solid Tumors v1.1.

**Table S2 The overall response rate in theresected population (n, %)**

|  | A1 (11) | A2 (10) | B1 (14) | B2 (11) |
| --- | --- | --- | --- | --- |
| CR | 1 (9.1) | 0 (0) | 1 (7.1) | 0 (0) |
| PR | 3 (27.3) | 7 (70.0) | 6 (42.9) | 8 (72.7) |
| SD | 4 (36.4) | 3 (30.0) | 6 (42.9) | 3 (27.3) |
| PD | 3 (27.3) | 0 (0) | 1 (7.1) | 0 (0) |
| NA | 0 (0) | 0 (0) | 0 (0) | 0 (0) |
| ORR | 4 (36.4) | 7 (70.0) | 7 (50.0) | 8 (72.7) |

CR, complete response; PR, partial response; SD, stable disease; PD, progressive disease; NA, notevaluable; ORR,a complete or partial response from baseline to the

presurgery scan per Response Evaluation Criteria in Solid Tumors v1.1.

**Table S3. Incidence of Immune-mediated Adverse Events associated with**

**neoadjuvant therapy (Safety Population) †**

| **Adverse event, %*** | A1 (N=12) | |  | A2+B1+B2 (N=40) | | |
| --- | --- | --- | --- | --- | --- | --- |
|  | All grades | Grade 3-4 |  | All grades | Grade3-4 | Grade 5 |
| Total AEs | 10 (83.3) | 4 (33.3) |  | 32 (80.0) | 14 (35.0) | 1 (2.5) |
| Any Grade AE ≥10% or all ‘Grade 3 ,4 or 5’ in either trial group | | | | | | |
| Exhausted | 7 (58.3) | 0(0) |  | 14 (35.0) | 1 (2.5) | 0(0) |
| Loss of appetite | 5 (41.7) | 0(0) |  | 12 (30.0) | 1 (2.5) | 0(0) |
| Decreased neutrophil count | 5 (41.7) | 0 (0) |  | 8 (20.0) | 4 (10.0) | 0(0) |
| Rash | 5 (41.7) | 0(0) |  | 6 (15.0) | 0(0) | 0(0) |
| Decreased white blood cell count | 4 (33.3) | 2 (16.7) |  | 7 (17.5) | 0(0) | 0(0) |
| Elevated AST | 4 (33.3) | 1 (8.3) |  | 7 (17.5) | 1 (2.5) | 0(0) |
| Elevated ALT | 3 (25.0) | 1 (8.3) |  | 11 (27.5) | 1 (2.5) | 0(0) |
| Hair loss | 3 (25.0) | 0(0) |  | 8 (20.0) | 0(0) | 0(0) |
| Decreased platelet count | 3 (25.0) | 0(0) |  | 4 (10.0) | 0(0) | 0(0) |
| Decreased TSH | 3 (25.0) | 0(0) |  | 0(0) | 0(0) | 0(0) |
| Elevated lipase | 2 (16.7) | 1 (8.3) |  | 1(2.5) | 0(0) | 0(0) |
| Anemia | 1 (8.3) | 0(0) |  | 14 (35.0) | 1 (2.5) | 0(0) |
| Elevated serum amylase | 1 (8.3) | 0(0) |  | 3(7.5) | 0(0) | 0(0) |
| Increased GGT | 1 (8.3) | 1 (8.3) |  | 3 (7.5) | 1 (2.5) | 0(0) |
| Hyperglycemia | 1 (8.3) | 0(0) |  | 4 (10.0) | 0(0) | 0(0) |
| Nausea | 0(0) | 0(0) |  | 9 (22.5) | 0(0) | 0(0) |
| Elevated conjugated bilirubin | 0(0) | 0(0) |  | 9 (22.5) | 1 (2.5) | 0(0) |
| Hyponatremia | 0(0) | 0(0) |  | 7 (17.5) | 0(0) | 0(0) |
| Hypoalbuminemia | 0(0) | 0(0) |  | 6 (15.0) | 0(0) | 0(0) |
| Diarrhea | 0(0) | 0(0) |  | 5 (12.5) | 1 (2.5) | 0(0) |
| Vomit | 0(0) | 0(0) |  | 5 (12.5) | 0(0) | 0(0) |
| Numb feet | 0(0) | 0(0) |  | 3 (7.5) | 0(0) | 0(0) |
| Decreased lymphocyte count | 0(0) | 0(0) |  | 3 (7.5) | 1 (2.5) | 0(0) |
| Itchy skin | 0(0) | 0(0) |  | 3 (7.5) | 0(0) | 0(0) |
| Increased total bilirubin | 0(0) | 0(0) |  | 3 (7.5) | 1 (2.5) | 0(0) |
| Immune-mediated enteritis | 0(0) | 0(0) |  | 1 (2.5) | 1 (2.5) | 0(0) |
| Bleeding hemorrhoids | 0(0) | 0(0) |  | 1 (2.5) | 1 (2.5) | 0(0) |
| Interstitial pneumonia | 0(0) | 0(0) |  | 0(0) | 0(0) | 1 (2.5) |

†Adverse events are listed in descending order of frequency.

ALT, alanine aminotransferase; AST, aspartate transaminase; GGT, γ-glutamyl

transpeptadase, TSH: Thyroid Stimulating Hormone,AE, adverse event.

*Events are listed in descending order of frequency in the total population

**Table S4 Characteristics of patients underwent ctDNA analysis**

| **A1 (7)** | **A2 (9) Total** | **B1 (13)** | **B2 (9)** | **Total** | ***p* value** |
| --- | --- | --- | --- | --- | --- |

**PD-L1 expression**

＜1%

1-49%

≥50%

**Sex**

Male

Female

**Age**

**(median, range)**

**Performance**

**status score**

0

1

**Pathological type**

Adenocarcinoma

Squamous

carcinoma

Adenosquamous

carcinoma

other

**Clinical Stage**

IIa

IIb

IIIa

IIIb

0 (0)

0 (0)

7 (100)

4 (57.1)

3 (42.9)

60 (50-71)

0 (0)

7 (100)

2 (28.6)

4 (57.1)

0 (0)

1 (14.3)

0 (0)

1 (14.3)

4 (57.1)

2 (28.6)

0 (0)

0 (0)

9 (100)

7 (77.8)

2 (22.2)

64 (43-73)

0 (0)

9 (100)

2 (22.2)

3 (33.3)

1 (11.1)

3 (33.3)

0 (0)

0 (0)

8 (88.9)

1 (11.1)

0 (0)

0 (0)

**16 (100)**

**11 (68.8)**

**5 (31.2)**

**60 (43-73)**

**0 (0)**

**16 (100)**

**4 (25.0)**

**7 (43.8)**

**1 (6.3)**

**4 (25.0)**

0 (0)

**1 (6.3)**

**12 (75.0)**

**3 (18.7)**

0 (0)

13 (100)

0 (0)

11 (84.6)

2 (15.4)

62 (22-71)

1 (7.7)

12 (92.3)

5 (38.5)

7 (53.8)

0 (0)

1 (7.7)

1 (7.7)

5 (38.5)

5 (38.5)

2 (15.4)

9 (100)

0 (0)

0 (0)

8 (88.9)

1 (11.1)

57 (48-71)

2 (22.2)

7 (77.8)

1 (11.1)

8 (88.9)

0 (0)

0 (0)

1 (11.1)

2(22.2)

4 (44.4)

2 (12.2)

**22 (40.9)**

**22 (59.1)**

**9 (75.0)**

**3 (25.0)**

**60 (22-71)**

**3 (13.6)**

**19 (86.4)**

**6 (27.3)**

**15 (68.2)**

0 (0)

**1 (4.5)**

**2 (9.1)**

**7 (31.8)**

**9 (40.9)**

**4 (18.2)**

-

0.534

>0.999

0.383

0.255

0.403

**T stage**

1 1 (12.3) 0 (0) **1 (6.3)** 0 (0) 0 (0) 0 ([0)](#bookmark10)

1c 1 (12.3) 1 (11.1) **2 (12.5)** 0 (0) 0 (0) 0 ([0)](#bookmark11)

2 0 (0) 1 (11.1) **1 (6.3)** 1 (7.7) 0 (0) **1 (4.5)**

0.909

2a 1 (12.3) 1 (11.1) **2 (12.5)** 3 (23.1) 2 (22.2) **5 (22.7)**

2b 1 (12.3) 0 (8.3) **1 (6.3)** 3 (23.1) 2 (22.2) **5 (22.7)**

3 3 (42.9) 4 (44.4) **7 (53.8)** 5 (38.5) 4 (44.4) **9 (40.9)**

4 0 (0) 2 (22.2) **2 (12.5)** 1 (7.7) 1 (11.1) **2 (9.1)**

**N stage**

0 0 (0) 0 (0) 0 (0) 3 (23.1) 1 (11.1) **4 (18.2)**

0.561

1 2 (28.6) 5 (55.6) **7 (43.8)** 5 (38.5) 5 (55.5) **10 (45.5)**

2 5 (71.4) 4 (44.4) **9 (56.2)** 5 (38.5) 3 (33.3) **8 (36.4)**

**M stage**

-

0 7 (100) 9 (100) **16 (100)** 13 (100) 9 (100) **22 (100)**

*p*-values were calculated using Fisher exact test, except forage (t-test). *p*-values <0.05

(in bold) were considered statistically significant.

**Table S5 ctDNA/MRD status at each timepoint**

|  | **Overall** | **A1** | **A2** | **B1** | **B2** |
| --- | --- | --- | --- | --- | --- |
| **PD-L1** |  | ≥ 50% | ≥ 50% | 1-49% | <1% |
| **T0 (+)** | 89.5% (34/38) | 14.7% (5/34) | 26.5% (9/34) | 32.3% (11/34) | 26.5% (9/34) |
| **T0 (-)** | 10.5% (4/38) | 50.0% (2/4) | 0% (0/4) | 50.0% (2/4) | 0% (0/4) |
| **T1 (-)** | 73.0% (27/37) | 7.4% (2/27) | 25.9% (7/27) | 48.1% (13/27) | 18.5% (5/27) |
| **T2 (+)** | 34.2% (13/38) | 38.5% (5/13) | 15.4% (2/13) | 23.1% (3/13) | 23.1% (3/13) |
| **T2 (-)** | 65.8% (25/38) | 8.0% (2/25) | 28.0% (7/25) | 40% (10/25) | 24.0% (6/25) |
| **T3 (-)** | 72.4% (21/29) | 14.3% (3/21) | 38.1% (8/21) | 23.9% (11/21) | 23.8% (5/21) |
| **T3 (+)** | 27.6% (8/29) | 50% (4/8) | 12.5% (1/8) | 0% (0/8) | 37.5% (3/8) |

T0, pretreatment; T1, before the third cycle of neoadjuvant treatment;

T2, after neoadjuvant treatment prior to surgery; T3, within one month after surgery

and prior toadjuvant treatment.

**3. Supplementary Figures**

**Figure S1 Patient Disposition**


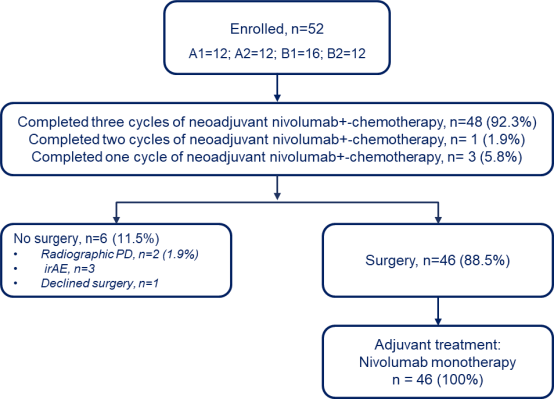


**Figure S2 Trial schema.**


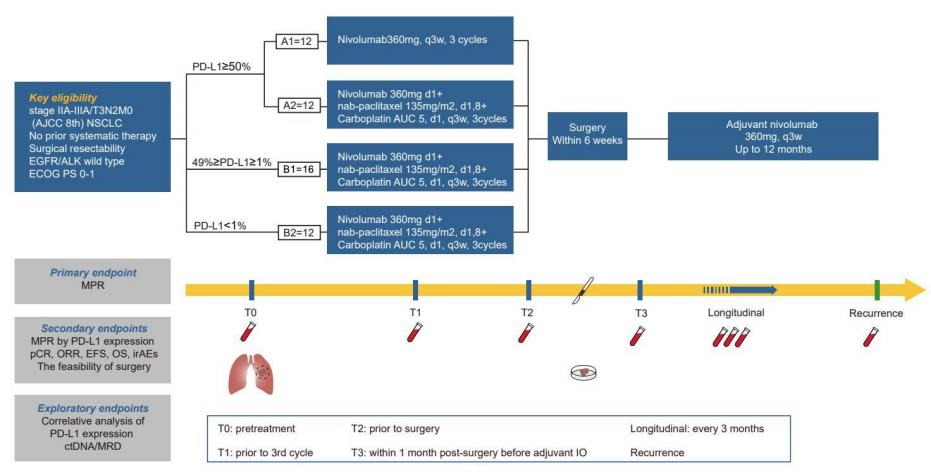

Supplement: Supplementary file 1 — Supplementary Information [file 41392_2023_1700_MOESM1_ESM.docx]
